# Supplementary material for: A multicenter study investigating the genetic analysis of childhood steroid-resistant nephrotic syndrome: Variants in COL4A5 may not be coincidental
Source: PLoS One. 2024 Dec 3;19(12):e0304864. doi: 10.1371/journal.pone.0304864 (PMC11614205; doi:10.1371/journal.pone.0304864)
Supplement: S1 Table — (PDF) [file pone.0304864.s001.pdf]

Patients/ID	Gene test	Age of onset (months)	Extra-kidney features	Prognosis
5	WT1	80	Mental retardation; Atrial septal	CKD5
38	PAX2	33	Atrial septal; Premature	Normal eGFR
61	COL4A5	147	Sensorineural deafness	CKD3
87	LMX1B	48	Nail—patella defect, Atrial septal defect;	CKD5
66	Negative	19	Atrial septal defect;	Nor eGFR
68	Negative	41	Autism	Normal eGFR
155	Negative	159	Mental retardation;	CKD5
257	Negative	124	Eczema	Normal eGFR
270	Negative	36	Mental retardation;	Normal eGFR
277	Negative	20	Mental retardation;	Normal eGFR
113	Negative	19	Oblique inguinal hernia	Normal eGFR
70	Negative	6	Mental retardation; Premature	Normal eGFR

S1 Table Extra-kidney manifestations of 12 SRNS. Mental retardation is the most frequent of these manifestations (5/12), followed by atrial septal defect (4/12). One patient had hearing loss at disease onset.
