# Supplementary material for: A multicenter study investigating the genetic analysis of childhood steroid-resistant nephrotic syndrome: Variants in COL4A5 may not be coincidental
Source: PLoS One. 2024 Dec 3;19(12):e0304864. doi: 10.1371/journal.pone.0304864 (PMC11614205; doi:10.1371/journal.pone.0304864)
Supplement: S1 Fig — (PDF) [file pone.0304864.s003.pdf]

Patients/ID	Gene test	Age of onset(Months)	Extra-kidney features	Prognosis
5	WT1	80	Mental retardation;Atrial septal	CKD5
38	PAX2	33	Atrial septal;Premature	Normal eGFR
61	COL4A5	147	Sensorineural deafness	CKD3
87	LMX1B	48	Nail—patella defect,Atrial septal defect;	CKD5
66	Nagtive	19	Atrial septal defect;	Nor eGFR
68	Nagtive	41	Autism	Normal eGFR
155	Nagtive	159	Mental retardation；	CKD5
257	Nagtive	124	Eczema	Normal eGFR
270	Nagtive	36	Mental retardation；	Normal eGFR
277	Nagtive	20	Mental retardation；	Normal eGFR
113	Nagtive	19	Oblique inguinal hernia	Normal eGFR
70	Nagtive	6	Mental retardation;Premature	Normal eGFR

Supplement Fig. S1 Extra-kidney manifestations of 12 SRNS,Mental retardation is most frequent(5/12),and follow by Atrial septal defect(4/12),only one had hearing loss at the initiate of disease
