# Supplementary material for: A multicenter study investigating the genetic analysis of childhood steroid-resistant nephrotic syndrome: Variants in COL4A5 may not be coincidental
Source: PLoS One. 2024 Dec 3;19(12):e0304864. doi: 10.1371/journal.pone.0304864 (PMC11614205; doi:10.1371/journal.pone.0304864)
Supplement: S2 Fig — (PDF) [file pone.0304864.s004.pdf]

Variable	Level	Univariate Cox regression analysis		Multivariate Cox regression analysis	
		HR (95%CI)	P-value	HR (95%CI)	P-value
Gene	no	reference		reference	
	yes	10.227 (3.505, 29.838)	<0.001	5.110 (1.122, 23.271)	0.035
Age		1.005 (0.994, 1.015)	0.377		
Sex	Male	reference			
	Female	1.321 (0.458, 3.807)	0.607		
Nation	Han	reference			
	Zhuang	1.578 (0.558, 4.459)	0.390		
	Others	3.320 (0.659, 16.729)	0.146		
Family history	no	reference		reference	
	yes	4.512 (1.657, 12.283)	0.003	1.597 (0.463, 5.507)	0.459
Extra-kidney manifestations					
Extra-kidney manifestations	no	reference		reference	
	yes	3.442 (1.087, 10.897)	0.036	1.082 (0.235, 4.984)	0.92
Resistant_style	not used	reference		reference	
	initial-resistant	0.187 (0.035, 0.987)	0.048	0.535 (0.041, 6.958)	0.633
	later-resistant	0.052 (0.006, 0.442)	0.007	0.376 (0.020, 7.242)	0.517
Upro24		1.000 (1.000, 1.000)	0.337		
Scr		1.002 (1.000, 1.004)	0.040	0.999 (0.994, 1.003)	0.545
eGFR		0.993 (0.984, 1.002)	0.140		
Hypertension	no	reference			
	yes	2.322 (0.859, 6.272)	0.097		
kidney biopsy	not done	reference			
	MCD	4.246 (0.524, 34.402)	0.176		
	FSGS	4.079 (0.446, 37.341)	0.213		
	MsPGN	2.196 (0.129, 37.370)	0.586		
	MN	0.000 (0.000, Inf)	0.998		
	DMS	6.576 (0.389, 111.250)	0.192		
Clinical style	data unavailable	reference			
	simplex	0.115 (0.012, 1.119)	0.062		
	nephritic	0.276 (0.031, 2.421)	0.245		
Response to immunosuppressant	not used	reference		reference	
	responder	0.109 (0.024, 0.493)	0.004	0.284 (0.022, 3.644)	0.334
	no-responder	0.715 (0.219, 2.337)	0.579	0.865 (0.085, 8.837)	0.903

HR: hazard ratio; CI: confidence interval

Supplement Fig. S2 Univariate Cox regression analysis with 2 group progression to CKD
