# Supplementary material for: A multicenter study investigating the genetic analysis of childhood steroid-resistant nephrotic syndrome: Variants in COL4A5 may not be coincidental
Source: PLoS One. 2024 Dec 3;19(12):e0304864. doi: 10.1371/journal.pone.0304864 (PMC11614205; doi:10.1371/journal.pone.0304864)
Supplement: Attachment — Submitted filename: Response to Reviewers.docx [file pone.0304864.s007.docx]

Dear Editors and Reviewers:

Thank you very much for your comments and recognition on the manuscript entitled “A multicenter study investigating the genetic analysis of childhood steroid-resistant nephrotic syndrome: variants in COL4A5 may not be coincidental”.

Based on your comment and request, we have made minor modification on the original manuscript, the funding sources were disclosed in the cover letter and their roles were specified in the manuscript. Any funding-related details have been removed from our manuscript. Here, we attached revised manuscript in doc for your approval. A document answering every question from the referees was also summarized and enclosed. Should you have any questions, please contact us without hesitate.

Those comments are all valuable and very helpful for revising and improving our paper, as well as the important guiding significance to our researches. We have studied comments carefully and have made correction which we hope meet with approval. The main corrections in the paper and the responds to the reviewer’s comments are as following:

Reviewer #1:

1. The word “renal” should be changed to “kidney”.

Respond: Thanks for your careful reminder, we have modified the word “renal” to “kidney”.

2. The sentences NS (massive proteinuria) probably an early phenotype of COL4A5 gene mutations (line 387) needs to be explained.

Respond: Thanks for your suggestion, the sentences “NS (massive proteinuria) probably an early phenotype of COL4A5 gene mutations” have explained to “In the early stages of COL4A5 gene variant, the primary clinical symptoms may encompass not just hematuria, also with massive proteinuria.”Line388-390.

3. Change the word “mutations” to “variants”.

Respond: Thanks for your suggestion, we have modified the word “mutations” to “variants”.

We tried our best to improve the manuscript and made some changes in the manuscript.  These changes will not influence the content and framework of the paper. And here we did not list all the changes but marked in highlight in revised paper. We appreciate for Editors/Reviewers’ warm work earnestly, and hope that the correction will meet with approval.

On behalf of my co-authors, we would like to expression our great appreciation to the editors and reviewers.

Yours sincerely,

Dr. Yuanhan,Qin,

Department of Pediatrics,

The First Hospital of Guangxi Medical University,

Nanning, Guangxi,China,

Tel: 86-13607819988

E-mail address:

[qinyuanhan2022@163.com](mailto:qinyuanhan2022@163.com)
